# Dysregulation of Lipid Metabolism Serves as A Link Between Alzheimer’s and Cardiovascular Disease, As Witnessed in A Cross-Sectional Study

**DOI:** 10.14336/AD.2024.0434

**Published:** 2025-05-31

**Authors:** Laura Mourino-Alvarez, Cristina Juarez-Alia, Tamara Sastre-Oliva, Inés Perales-Sánchez, German Hernandez-Fernandez, Eduardo Chicano-Galvez, Ángela Peralbo-Molina, Felipe Madruga, Emilio Blanco-Lopez, Teresa Tejerina, María G. Barderas

**Affiliations:** ^1^Department of Vascular Physiopathology, Hospital Nacional de Paraplejicos, SESCAM, 45071 Toledo, Spain.; ^2^Department of Vascular Physiopathology, Hospital Nacional de Paraplejicos, IDISCAM, 45071 Toledo, Spain.; ^3^IMIBIC Mass Spectrometry and Molecular Imaging Unit, Maimonides Biomedical Research Institute of Cordoba (IMIBIC), Reina Sofia University Hospital, University of Cordoba (UCO), Córdoba, Spain.; ^4^Departament of Geriatrics, Hospital Virgen del Valle, SESCAM, Toledo, Spain.; ^5^Department of Cardiology, Ciudad Real General University Hospital, Ciudad Real, Spain.; ^6^Department of Pharmacology, School of Medicine, Universidad Complutense de Madrid, Madrid, Spain.

**Keywords:** dementia, Alzheimer, cardiovascular disease, lipid metabolism, biomarkers

## Abstract

Cardiovascular risk factors and established cardiovascular disease (CVD) increase the risk of suffering dementia of the Alzheimer's type (DAT). Here, we set out to define specific molecular profiles of CVD in patients with DAT to better understand its relationship, to unravel the mechanisms underlying the high risk of developing DAT in CVD patients and to define new markers of early disease. Plasma samples from patients with DAT, with and without CVD, were analyzed through a multiomics approach, with integration of metabolomics and proteomics datasets using the OmicsNet web-based tool. Metabolomics results showed an enrichment in lipids and lipid-like molecules. Similarly, the most significant cluster identified through proteomics was formed by 5 proteins related to lipoprotein and cholesterol metabolism. After integration and functional enrichment, glycerolipid metabolism, fatty acid degradation and sphingolipid metabolism were among the most significant functions. Finally, the differential expression of ABCA1 and APOH proteins was verified, in an independent cohort also including controls and patients with CVD alone. Both proteins positively correlated with phospho-Tau (181), a classical hallmark of DAT. Different molecular profiles exist in patients with DAT, with and without CVD, with exacerbated alterations in patients in which DAT and CVD co-exist. This information may help to define biomarkers like ABCA1 and APOH that identify patients with cardiovascular dysfunction that are at high risk of developing DAT. Such markers will allow more personalized interventions to be selected, a further step towards precision medicine for individuals whose molecular profiles indicate a distinct response to the same management strategies.

## INTRODUCTION

Neurodegenerative Dementia of the Alzheimer's type (DAT) is the most common cause of dementia and a global health problem, with a significant influence on family and society [1]. Although dementia is an age-related neurodegenerative disease, it is not inevitable [2-4]. In fact, 12 factors have been described that can modify this outcome: lower levels of education, hearing impairment, depression, physical inactivity, limited social contact, excessive alcohol consumption, traumatic brain injury, air pollution and different cardiovascular risk factors like the presence of hypertension, diabetes, obesity or a smoking habit. Tackling these risk factors could prevent around 40% of dementias worldwide, reducing neuropathological damage and enhancing the cognitive reserve [2, 3].

Established cardiovascular disease (CVD), such as heart failure, coronary artery disease (CAD), aortic valve stenosis or atrial fibrillation, has been identified as a risk factor for the development of all types of dementia, including DAT [5, 6]. For example, subjects with atrial fibrillation present a high risk of developing Alzheimer’s disease (AD), although the mechanisms implicated remain unclear [7-9]. Heart failure is also associated with cognitive impairment and AD, possibly because it results in hypoperfusion of the brain [10]. In addition, thrombo-embolic factors and systemic responses to heart failure may contribute to cognitive dysfunction [10]. Regarding CAD, it is associated with the levels of circulating amyloid-β (Αβ) in adults, regardless of the presence of DAT [11-13].

It was recently suggested that a 10% reduction in the prevalence of cardiovascular risk factors could prevent more than nine million dementia cases worldwide by 2050 [4, 14]. Lipid metabolism plays an important role in both DAT and CVD, including alterations to lipoproteins and other lipid related proteins like fatty acid binding proteins [15, 16]. Indeed, different lipid classes experience changes in the brain, cerebrospinal fluid (CSF) and blood of patients with dementia, such as phospholipids, phosphatidylcholines, sphingolipids, and sterols [17, 18]. Moreover, plasma cholesterol may damage the integrity of the blood-brain barrier (BBB), altering the clearance of Αβ in the brain and even enhancing its production [19].

Better understanding the molecular mechanisms implicated in the development of CVD in patients with AD is crucial to delaying these pathologies, in particular through earlier diagnosis, prevention strategies and novel targeted therapies. With that in mind, we followed an untargeted mass spectrometry (MS)-based metabolomics and proteomics workflow, which included knowledge-driven integration through the use of different bioinformatics tools. In a cohort of subjects with DAT, we identified different molecular profiles based on the presence or absence of CVD, highlighting lipid metabolism as a key process. Defining these profiles paves the way to design new means to manage CVD in subjects with DAT, and it may well enable the identification of patients with CVD who present a higher risk of developing DAT. Accordingly, novel, and more effective personalized preventive measures could be implemented to reduce the burden of these two pathologies.

## MATERIALS AND METHODS

### Patient selection

Patients were enrolled at the Cardiology and Geriatric Services of the Hospital Universitario of Toledo, and they were assigned to one of the four study groups: i) healthy subjects; ii) patients with CVD; iii) patients with DAT; and iv) patients with DAT and CVD. The study was conducted in accordance with the Declaration of Helsinki and approved by the Ethics Committee of Hospital “Virgen de la Salud”. Informed consent was obtained from all subjects involved in the study.

**Table 1:** Clinical characteristics of the participants.

	Discovery phase				Verification phase			
	DAT (n=5)	DAT+CVD (n=5)	*p*	C (n=7)	CVD (n=7)	DAT (n=7)	DAT+CVD (n=7)	*p*
**Age, years**	82.6±6.6	85.8±4.2	0.385	83.6±5.8	85.6±5.4	85.6±7.0	89.3±7.9	0.448
**Sex, M/F**	1/4	0/5	0.292	2/5	2/5	3/4	2/5	0.921
**AHT, n**	5	5	-	5	4	3	6	0.375
**Dyslipidemia, n**	2	1	0.49	6	4	2	2	0.099
**Diabetes, n**	0	0	-	0	1	0	1	0.541
**Obesity, n**	0	1	0.292	1	2	0	2	0.444
**Heart failure, n**	0	4	0.01	0	2	0	0	0.091
**IHD, n**	0	0	-	0	2	0	1	0.25
**Atrial fibrillation, n**	0	3	0.038	0	3	0	3	0.054
**PVD, n**	0	1	0.292	0	0	0	1	0.375
**Valve disease, n**	0	1	0.292	0	2	0	0	0.118

Abbreviations: C, healthy control subjects; CVD, cardiovascular disease; DAT, dementia of the Alzheimer's type; DAT+CVD, dementia of the Alzheimer's type with cardiovascular disease; AHT, Arterial Hypertension; IHD, ischemic heart disease; M/F, male/female; n, number of subjects with the corresponding clinical characteristic; PVD, Peripheral vascular disease; *p*, *p*-value.

Peripheral blood samples were collected from each participant in tubes containing EDTA, centrifuged for 15 min at 1,125 g and the resulting supernatant was immediately frozen at -80 °C until analysis. CVD was considered as heart failure, ischemic heart disease, atrial fibrillation, peripheral vascular disease, or valve disease. Patients with vascular dementia, Lewy body disease or frontotemporal dementia were excluded from the study. When distributing the participants into the four study groups significant differences in terms of age and gender were avoided. The discovery phase of both the proteomics and metabolomics analyses was carried out on a sample of 10 patients with DAT, 5 of whom also had CVD. The results were analyzed using different bioinformatics tools and selected proteins were verified in an independent cohort of 28 patients distributed among the four study groups (Table 1).

### Metabolomics workflow for the discovery phase

To remove proteins, and for metabolite extraction, the plasma was incubated with methanol for 15 min at 4 °C, and after two consecutive centrifugations the supernatant was recovered and transferred to a glass vial for analysis. A Quality Control (QC) was prepared by mixing the same volume of each analytical sample. Serial diluted QCs were also prepared (at 100%-80%-60%-40%-20%). Liquid chromatography (LC) separation was achieved using an Elute UHPLC (Bruker Daltonics, Germany), with mobile phase A (Water) and B (acetonitrile - ACN), both containing 0.1% formic acid (FA). LC involved a 30-minute run time, including a column wash following the gradient: 1% B 2 min, flow rate 0.25 mL/min; 99% B 15 min, flow rate 0.25 mL/min; 99% B 3 min, flow rate 0.25 mL/min; 1% B 0.1 min, flow rate 0.35 mL/min; 1% B 8.2 min, flow rate 0.35 mL/min; and 1% B 1.7 min, flow rate 0.25 mL/min. The autosampler was maintained at 4 °C throughout the analysis. Samples were randomly analyzed by triplicates, including a QC analysis every 6 runs. Samples were injected onto the column in a 5 µL volume using the μl Pick-up mode. A T-ReX Elute M-column Kit (Bruker Daltonics, Germany) was used for separation, maintained at 35 °C. This kit includes an Intensity Solo HPLC Column C18 (100 × 2 mm × 2 μm) coupled to a VanGuard pre-column.

MS data was acquired using a TIMS-TOF Pro instrument (Bruker Daltonics, Germany) operating in the 4D PASEF mode, and in both positive and negative ion modes. The parameters for MS acquisition were: capillary voltages at +4500 V and -3500 V for positive and negative ionization modes, respectively; dry gas, nitrogen at a rate of 9 L/min and at a dry temperature of 220 °C; nebulizer pressure, 4 bar; peak detection threshold, 100 counts; scan mode, PASEF with the mass scan range of 20 to 1300 Da for both MS and MS2 acquisition; acquisition cycle, 0.1 s with a mobility scan range of 0.45 to 1.45 Vs/cm^2^ in the positive mode and 0.45 to 1.30 Vs/cm^2^ in the negative mode; collision energy, 10 eV in both positive and negative ion modes. TIMS and mass calibration of the instrument were performed weekly using peaks from the Agilent ESI LC-MS tuning mix. An online recalibration of the data was carried out immediately after each sample acquisition using a mixture of Agilent ESI LC-MS tune mix and 10 mM sodium formate (1:1), injected directly into the ESI source via a syringe pump. The 20-minute LC-MS run time was divided into three segments for this purpose, with the switch between segments achieved using a conventional 6-port divert valve. The list of the peaks used for recalibration is presented in Table SM1 and SM2.

The data was initially evaluated with DataAnalysis 6.1 software (Bruker Daltonics) and the untargeted data treatment was performed using MetaboScape 8.0.2 software (Bruker Daltonics), compatible with the LC-TIMS-QTOF data acquired in our study. The Time-aligned Region complete eXtraction (T-ReX) algorithm, a feature finder embedded in MetaboScape, was utilized for data preprocessing. This included mass calibration, peak picking, time alignment and within-batch correction. The within-batch correction was performed by using those molecular entities present in all QCs, and filtering by variance across QCs, keeping only those which exhibited a maximum variance of 40% or 20% before and after correction, respectively. Quality control (QC) samples were also analyzed, and feature merging was also performed in the MetaboScape interface. The features detected in both polarities during the peak-picking step were merged into unified features to avoid any overlap in the statistical evaluation. To mitigate the impact of missing values (features with zero intensity), the T-ReX algorithm's automatic region complete feature extraction was employed. This algorithm detects missing features or those declared as features of zero intensity but present in other samples of the same group (e.g., below the peak-picking intensity threshold), and it automatically re-extracts the feature in a targeted manner. Finally, a homemade R-script was used to filtered data from MetaboScape, considering only features that presents an R2 above 0.8 when representing the intensity of each molecular feature in serial diluted QCs analysis.

### Proteomics workflow in the discovery phase

The total protein in the plasma samples was first quantified by microfluorimetry (Qubit™ Protein Assay), and the protein extracts were then manually digested with Trypsin and LysC in an advanced in-Stage-Tip (iST) dual digestion. The digested samples were diluted to a final concentration of 10 ng/µl in LC-MS Water + 0.1% FA (v/v) and loaded into Evotips Pure (200 ng/tip). Subsequently, the LC and MS analysis was performed using an EvosepOne nanoLC (Evosep, Odense, Denmark, 2021) coupled to a TIMSTOF-Flex (Bruker Daltonics, Bremen, Germany, 2022). Two main acquisition methods were used: DDA (Data Dependent Analysis) acquisition for DIA (Data Independent Analysis) method adjustment; and DIA for the final data acquisition in both PASEF and PaSER modes to ensure correct data acquisition. The DDA-PASEF runs acquired from a pool of all the samples obtained in the study were used to generate a specific spectral library with MSFragger through its graphical interface Fragpipe (https://fragpipe.nesvilab.org), employing the default parameters. The fasta file for the Human Reference Proteome (March 2022 version, www.uniprot.org/taxonomy/9606) was downloaded from Uniprot (https://uniprot.org) and used as a search database.

For the DIA-PASEF approach, the mass range was defined from 352.5 m/z to 1199.6 m/z and the ion mobility range was set from 0.7 1/K0 to 1.3 1/K0. The collision energy was decreased linearly from 59 eV at 1/K0 = 1.60 Vs cm^-2^ to 20 eV at 1/K0 = 0.60 Vs cm^-2^, and the cycle time was estimated by the system as 1.38 seconds (the detailed optimized method is shown in Table SM3). The MS analysis of the samples was performed randomly to prevent any batch effect and a pool of the entire experiment was used to control the system’s stability during the entire DIA-PASEF acquisition (QC sample). Spectronaut (Biognosys) software was used to identify and quantify the complete set of samples.

### Bioinformatics workflow

Both the proteomics and metabolomics sets were evaluated using MetaboAnalyst V.6.0 software [20]. On the one hand, the normalized proteomics data was log transformed and pareto scaled. On the other hand, the normalized metabolomics data was square root transformed and pareto scaled. In both cases, missing values were replaced by 1/5 of the minimum positive value for each variable. A Partial Least-Squares Discriminant Analysis (PLS-DA), the variable importance in projection (VIP) score, hierarchical clustering, fold change (FC) calculation and statistical t-test were performed on the normalized data. Statistical significance was obtained through the t-test and considered for features with a corrected FDR *p*-value <0.05. The differentially expressed proteins (p-value <0.05, FC >1.5) were then used as the input for the web based Search Tool for the Retrieval of Interacting Genes/Proteins (STRING v12.0), in order to generate protein-protein interaction (PPI) networks [21]. These were also used for the functional analysis, while metabolites with a VIP score >1 were annotated and classify according to Kyoto Encyclopedia of Genes and Genomes (KEGG), Human Metabolome Database (HMDB), LipidMaps and ClassyFire.

Our two omics datasets were integrated with OmicsNet 2.0. software for network visualization and functional enrichment analysis [22]. First, the PPIs and protein-metabolite interactions were used for subnetwork building using Innate DB and Recon3D as databases. Over-representation analysis was performed using all the nodules (proteins and metabolites) against the KEGG database.

### Immunodetection for verification

Plasma samples were obtained from an independent cohort of patients for the four study groups. Equal amounts of protein (25 µg) from each patient were resolved by SDS-polyacrylamide gel electrophoresis (SDS-PAGE) in a Bio-Rad Miniprotean II electrophoresis cell run at a constant current of 25 mA/gel. After electrophoresis, the proteins were transferred to a nitrocellulose membrane under a constant voltage of 20 V for 40 min and the membranes were stained with Ponceau S to ensure an equal amount of protein was loaded for each patient. The membranes were then blocked for 1 h and probed overnight with the corresponding primary antibody (Table SM4): a rabbit polyclonal antiserum raised against Apolipoprotein H (APOH, 1/5000: Abcam, ab108348); ATP-binding cassette sub-family A member 1 (ABCA1, 1/500: Abcam, ab307536); and tau phosphorylated at residue 181 (p-Tau(181), 1/250: Cell Signaling, #12885). After washing, the membranes were incubated with a specific HRP-conjugated secondary antibody (Abcam, ab205718, 1:2000 for APOH and Cell signaling #7074P2, 1:1000 for ABCA1 and p-Tau(181)) and antibody binding was detected by enhanced chemiluminescence (ECL: GE Healthcare), according to the manufacturers’ instructions. Densitometry was performed with the ImageQuantTL software (GE Healthcare, IL, United States).

### Statistical analysis

Statistical analyses were performed using JASP v.0.18.1, an open-source statistical analysis software. Initially, normality was assessed with the Kolmogorov-Smirnov test and consequently, normally distributed variables were analyzed using parametric tests and those distributed non-normally were analyzed with non-parametric tests. The differences between the groups for the clinical parameters with discrete variables were calculated using a chi-squared test, while continuous variables were calculated with one-way ANOVA and a Tukey’s *post hoc* analysis for the four group comparisons when data is normally distributed and, with Kruskal-Wallis when it is not. The descriptive data was presented as the mean ±standard deviation (SD) or as the number of subjects presenting a given condition. Statistical significance was accepted when *p*-value <0.05 and ROC curves were generated using SPSS 15.0 for windows software (SPSS Inc.).

**Figure 1.**
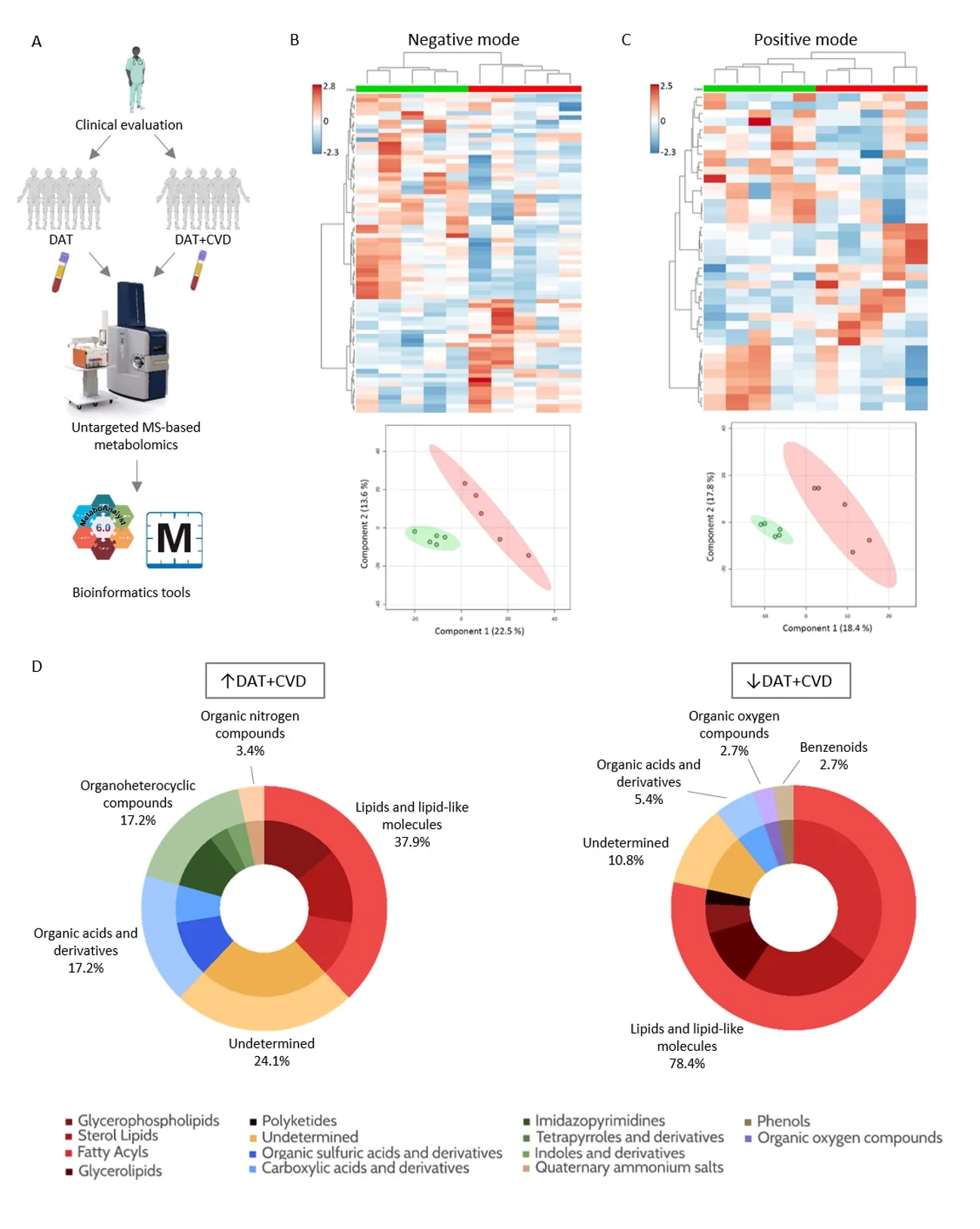
**Untargeted metabolomics profiling of plasma simple from patients with DAT, with and without CVD (n=5 subjects/group)**. (**A**) Clinical sample collection and processing. (**B**) Heat map and PLS-DA plot of negative mode acquisition showing the separation of the study groups: DAT patients in green, DAT+CVD patients in red. (**C**) Heat map and PLS-DA plot of positive mode acquisition showing the separation of the two study groups: DAT patients in green, DAT+CVD patients in red. (**D**) Classification of the metabolites identified according to their chemical structure.

## RESULTS

This study set out to identify a molecular profile specific to DAT patients with concomitant CVD, with a view to unraveling the effect of CVD on the development of DAT and to define new biomarkers of this disease. This latter goal will help improve the clinical management of these patients at neurological and cardiology units, taking a step towards improving personalized precision medicine.

**Figure 2.**
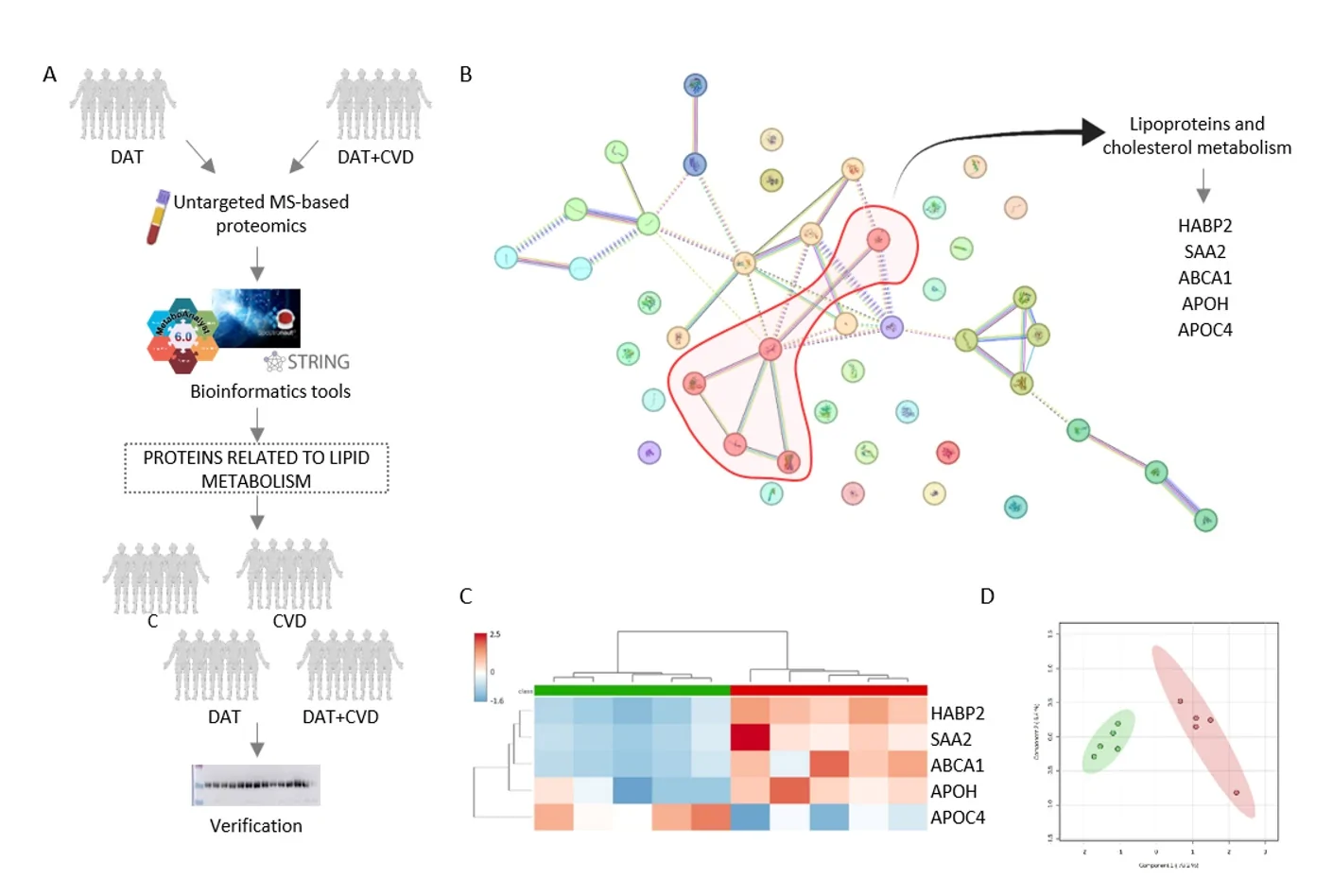
**Untargeted proteomic profiling of plasma samples from patients with DAT, with and without CVD (n=5 subjects/group)**. (**A**) Clinical sample collection and processing, including the bioinformatics analysis and selection of the most promising candidates for verification. (**B**) Protein-protein interaction (PPI) networks, highlighting the cluster made up of HABP2, SAA2, ABCA1, APOH and APOC4, that is related to lipoproteins and cholesterol metabolism. (**C**) Heat map of the five proteins related to lipoproteins and cholesterol metabolism: DAT patients in green, DAT+CVD patients in red. (**D**) PLS-DA plot showing the separation of the 2 study groups: DAT patients in green, DAT+CVD patients in red.

The untargeted metabolomics analysis revealed 112 molecular features with a VIP value >1.0, 73 in negative mode and 39 in positive mode. Hierarchical clustering and the PLS-DA showed a perfect separation of the two groups of study, discriminating patients with and without CVD (Fig. 1B and C). Of these, 66 molecular features were identified with differences between patients with and without CVD, 37 of which were lower and 29 higher in patients with DAT and CVD, and these metabolites were classified according to their types (Supplementary Table 5). Accordingly, we found that 40 compounds (60.6%) were lipids and lipid-like molecules, including fatty acyls (40 %), sterol lipids (32.5 %), glycerophospholipids (15 %), glycerolipids (10 %), polyketides (2.5 %) and prenol lipids (2.5 %). We also found different organic acids and derivatives (7 compounds, 10.7 %), organoheterocyclic compounds (5 compounds, 7.6%), organic oxygen compounds, organic nitrogen compounds and benzenoids (one from each group, 1.5 % each). Importantly, 11 compounds (16.7 %) could not be accurately classified due to a lack of information (Fig. 2C).

After the proteomics and statistical analysis, we performed a functional ontology enrichment and PPI analysis to deepen the implicated molecular pathways and their relationship with the metabolomics results. This analysis highlighted the existence of an enriched cluster related to lipoprotein and cholesterol metabolism, which was formed of 18 terms related to lipid metabolism (Table 2). The strong relationship between this cluster and our metabolomics results encouraged us to focus our work on these proteins. This cluster displays PPI enrichment (*p*-value=1.02E-9), and it was made up of ABCA1, APOH, apolipoprotein C-IV (APOC4), hyaluronan-binding protein 2 (HABP2) and serum amyloid A-2 protein (SAA2: Fig. 2B). While there was an increase in ABCA1, APOH, HABP2 and SAA2, APOC4 decreased in patients with DAT and CVD relative to the patients with CVD alone, as evident in the heat-map (Fig. 2C). In addition, as well as the PLS-DA analysis (Fig. 2D), this hierarchical clustering showed that these 5 proteins can discern the presence of CVD in DAT patients.

**Table 2:** Terms that define the cluster related to lipoproteins and cholesterol metabolism: FDR, false discovery rate.

TERM ID	TERM DESCRIPTION	FDR
**GOCC:0034364**	High-density lipoprotein particle	8.49E-08
**GO:0034364**	High-density lipoprotein particle	7.95E-05
**CL:18726**	Complement and coagulation cascades, and Protein-lipid complexes	0.00011
**CL:18960**	High-density lipoprotein particle	0.00011
**WP5323**	Fatty Acid and Lipoprotein Transport in Hepatocytes	0.00055
**CL:18966**	High-density lipoprotein particle	0.0045
**GO:0034361**	Very-low-density lipoprotein particle	0.0049
**GOCC:0005576**	Extracellular region	0.0055
**GOCC:0034361**	Very-low-density lipoprotein particle	0.0055
**hsa04979**	Cholesterol metabolism	0.0211
**HSA-8963898**	Plasma lipoprotein assembly	0.0247
**WP1533**	Vitamin B12 metabolism	0.0265
**HP:0002730**	Chronic non-infectious lymphadenopathy	0.0283
**WP15**	Selenium micronutrient network	0.0312
**WP176**	Folate metabolism	0.0312
**WP5304**	Cholesterol metabolism	0.0312
**BTO:0000759**	Liver	0.0357
**HSA-9029569**	NR1H3 & NR1H2 regulate gene expression linked to cholesterol transport and efflux	0.0412

### Integrating Metabolome and Proteome data

**Figure 3.**
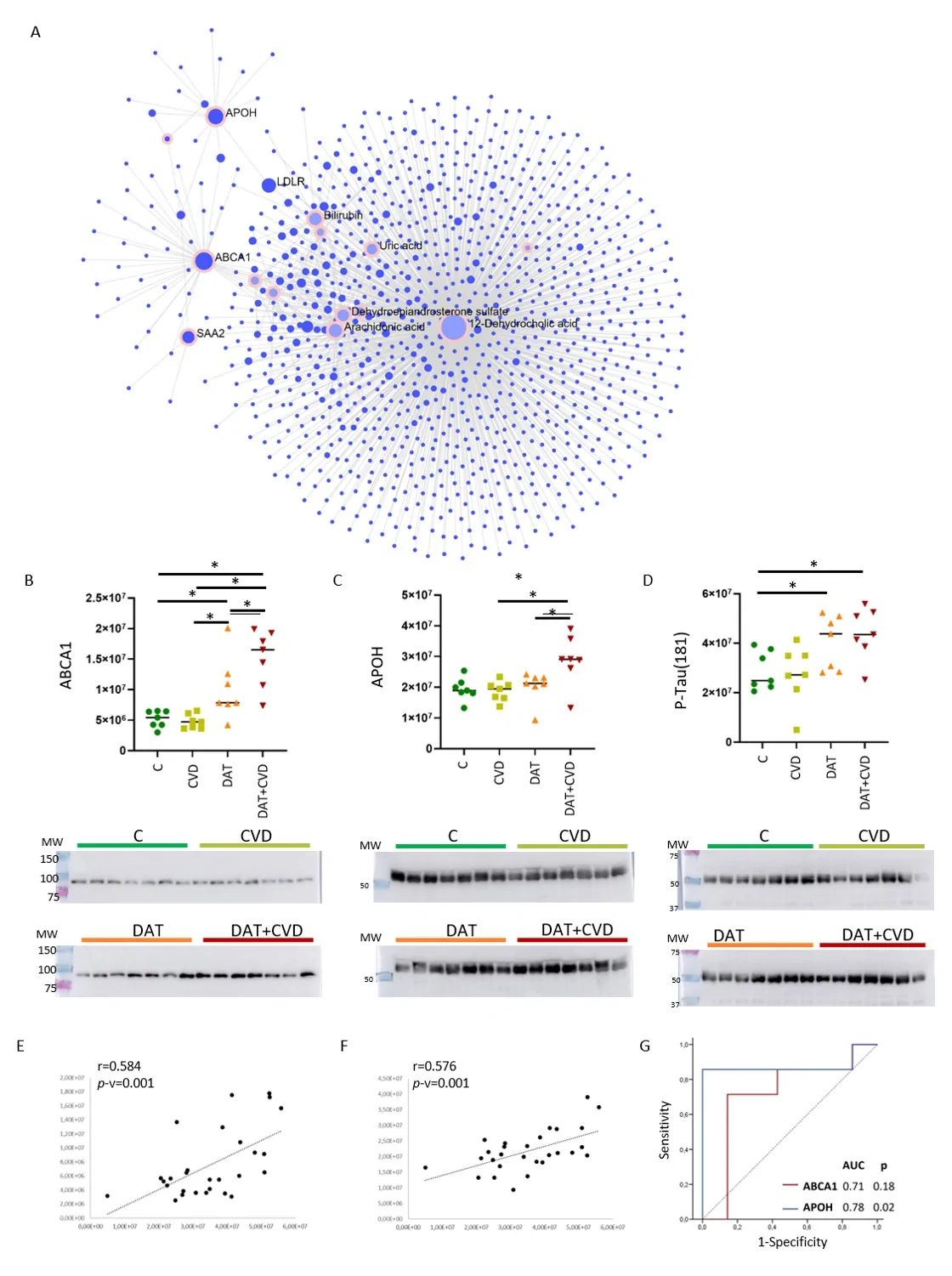
**Integration of the proteomics and the metabolomics results, and verification of ABCA1 and APOH**. (**A**) A multiomics network presenting the relationship between the metabolic and proteomic profiles. (B, C) A verification of ABCA1 and APOH showed increased levels of both proteins in patients with DAT and CVD (n=7 subjects/group). (**D**) Immunodetection of p-Tau(181), a hallmark of Alzheimer’s disease (n=7 subjects/group). (E, F) Correlation plots between ABCA1 (E) and APOH (F) with p-Tau(181). (**G**) ROC curves of ABCA1 and APOH to assess the sensitivity and specificity of these potential markers. The discriminative power of APOH is higher than that of ABCA1. Abbreviations: AUC, area under the curve.

It is becoming increasingly evident that integrated analyses across multiple ‘omics’ platforms are necessary to interrogate complex biological systems. Accordingly, OmicsNet was used to integrate the ‘omics’ data obtained, mapping the interactome network between the 5 proteins related to lipoprotein and cholesterol metabolism, as well as the metabolites with VIP >1. The PPI network was formed by 57 nodes and 59 edges, while the metabolite-protein network had 1034 nodes and 1188 edges. The final subnetwork consisted of 10 metabolites, 1076 proteins and 1247 edges (Fig. 3A). Two of the proteins of interest appeared among the top ten compounds with strongest betweenness and highest degree: ABCA1 and APOH. The functional analysis of this subnetwork using the KEGG database of proteins and metabolites together showed an enrichment in several pathways related to lipids. Within the top 15 of the most significant functions, we found glycerolipid metabolism, fatty acid degradation and sphingolipid metabolism (Table 3), highlighting the importance of lipid compounds in the relationship between CVD and DAT.

**Table 3:** **Functional enrichment according to the KEGG database**. The integrative *p*-value combining metabolites and proteins is shown, and the functions related to lipid metabolism are in bold.

Pathway	Total hits	Integrative *p*-value
Metabolism of xenobiotics by cytochrome P450	64	2.38E-49
**Glycerolipid metabolism**	56	6.11E-48
Drug metabolism - cytochrome P450	58	1.29E-42
**Fatty acid degradation**	41	5.79E-36
**Sphingolipid metabolism**	40	1.62E-31
**Retinol metabolism**	47	6.04E-30
Alzheimer disease	35	8.57E-28
**Ether lipid metabolism**	37	7.47E-27
**alpha-Linolenic acid metabolism**	25	1.51E-24
N-Glycan biosynthesis	36	7.95E-24
Glycosylphosphatidylinositol (GPI)-anchor biosynthesis	24	3.06E-22
Drug metabolism - other enzymes	44	5.06E-22
**Fatty acid elongation**	24	2.86E-20
Ascorbate and aldarate metabolism	23	1.39E-18
Glycosaminoglycan biosynthesis - heparan sulfate / heparin	21	1.48E-17

### Immunodetection

The relevance of the two proteins identified was confirmed in Western Blots: ABCA1 and APOH (Table 4). In both cases, more of these proteins were found in patients with DAT and CVD relative to the other 3 study groups, including patients with DAT (Fig. 3B and C). In addition, tau phosphorylated at residue 181 (p-tau181) was also analyzed by immunodetection, confirming the higher levels of this common marker of AD in patients with DAT, both with and without CVD (Fig. 3D). Importantly, the levels of this marker were positively correlated with those of ABCA1 (r=0.584; p-value=0.001: Fig. 3E) and APOH (r=0.576; p-value=0.001: Fig. 3F).

**Table 4:** **Western blot densitometry data expressed as arbitrary units**. Mean values of ABCA1, APOH and p-Tau(181) are shown.

	C	ECV	DAT	DAT+ECV	*p*-value
**ABCA1**	4.43x10^6^±1.24x10^6^	4.03x10^6^±9.87x10^5^	8.78x10^6^±4.60x10^6^	1.32x10^7^±4.26x10^6^	0.000
**APOH**	1.94x10^7^±3.67x10^6^	1.87x10^7^±3.22x10^6^	2.04x10^7^±5.05x10^6^	2.88x10^7^±8.18x10^6^	0.012
**p-Tau(181)**	2.89x10^7^±7.84x10^6^	2.74x10^7^±1.19x10^7^	4.04x10^7^±1.09x10^7^	4.40x10^7^±1.04x10^7^	0.006

To assess the sensitivity and specificity of ABCA1 and APOH as biomarkers, ROC curves were calculated from the immunodetection data. APOH had greater diagnostic power than ABCA1 in relation to patients with DAT with and without CVD (APOH-AUC=0.878, *p*-value=0.018; ABCA1-AUC=0.714, *p*-value=0.18: Figure 3G). Interestingly, either of these proteins was able to discriminate between controls and patients with DAT (APOH-AUC=0.531, *p*-value=0.848; ABCA1-AUC= 0.633, *p*-value=0.406). As expected, p-Tau(181) was not able to discriminate the presence of DAT, regardless the existence of CVD (AUC<0.6 and *p*-value>0.6 in both cases).

## DISCUSSION

Both DAT and CVD are highly prevalent pathologies, often occurring together in the elderly. The root cause of DAT remains unknown, despite the huge efforts into the study of this pathology, perhaps due to its complex and multifactorial etiology, with senile plaques and neurofibrillary tangles as key hallmarks [23]. The role of CVD in DAT has recently gained importance, with the relationship between both pathologies well established and significantly, different therapeutic options available to manage CVD [24]. Indeed, about 25% of DAT cases are attributable to cardiovascular risk factors and therefore, they could potentially be prevented by tackling these risk factors. As such, it is essential to gain a better understanding of the molecular pathways involved in the development of CVD in the context of DAT. This will facilitate the choice of better treatments for these patients with a view to preventing systemic damage and to also delay cognitive decline. Here we investigated alterations to the proteome and metabolome of patients with DAT, both with and without CVD, using state-of-the-art MS techniques. These results were analyzed using different bioinformatics tools to extend the biological relevance of our findings.

**Figure 4.**
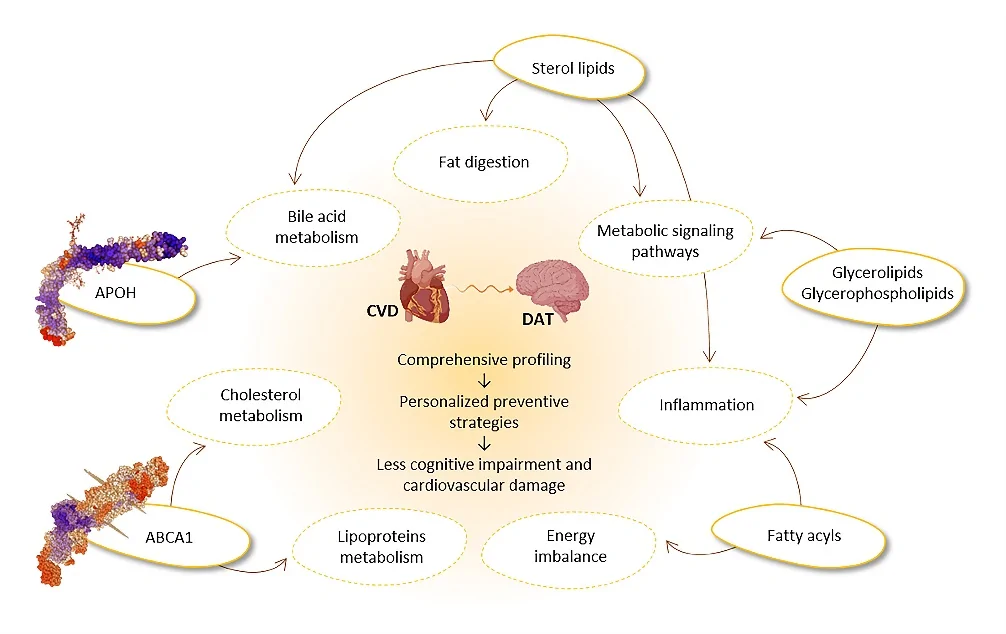
**Overview of the molecular pathways related to lipids metabolism that have a role in the relation between DAT and CVD**. A comprehensive profiling of the different implied molecules is essential for improving disease management through the development of new personalized preventive strategies. The final aim is to anticipate cognitive impairment and cardiovascular damage, reducing the burning of these pathologies.

Lipids have been highlighted as important effectors in DAT and CVD. Clinical, epidemiological and animal studies suggest that an imbalance in lipid metabolism is a risk factor for both DAT [25-27] and for CVD [27]. As we also found differences in the amounts of numerous lipids between DAT and DAT+CVD, we focused on this class of compounds, in particular fatty acyls, sterol lipids, glycerophospholipids and glycerolipids, the groups in which the most significant differences were found. Finally, we addressed the importance of ABCA1 and APOH in relation to lipid metabolisms in DAT patients with CVD (Fig. 4).

There are tens of thousands of fatty acyl lipid species, characterized by their molecular structure of a hydrocarbon chain with a carboxylic group. This is a very varied group, as the carboxyl group can be linked to a diverse range of substituents and the hydrocarbon chain can contain double bonds, adding further options to their simple variable length and branched or linear nature. Among the fatty acyls, we have found alterations to fatty acids, the most prominent fatty acyls. Unsaturated fatty acids are among the lipids most often diminished in patients with DAT and CVD, and fatty esters are also commonly affected, 5 of which decrease and 3 increase in patients with DAT and CVD. There are several studies demonstrating the potential neuroprotective role of unsaturated fatty acids [28-31], with a decrease in these compounds associated with a high risk of AD [32, 33].

Abnormal fatty acid metabolism is also an important cardiovascular risk factor. Monounsaturated fatty acids like palmitoleic acid are involved in many physiological processes related to CVD, including monocyte recruitment and the release of pro-inflammatory cytokines like tumor necrosis factor-α (TNF-α) and interleukin-6 [34, 35]. Although their role remains controversial, they probably produce different effects depending on the isomer, and the organ or disease model under study [36]. Regarding polyunsaturated fatty acids, a special class of fatty acids that contain multiple double bonds, many studies are ongoing into their role in the prevention and treatment of major cardiovascular events [37, 38]. Many such fatty acids, e.g. linolenic acid (C18:3) or docosahexaenoic acid (C22:6), have been proposed to influence cardiovascular risk factors like dyslipo-proteinemia and hypertension [39-41], and to exert anti-inflammatory effects [42-44]. Nevertheless, it remains unclear whether these compounds are pro- or anti-inflammatory [45]. For example, arachidonic acid (C20:4) may acts as an inflammatory mediator and induce oxidative stress [46, 47].

We also found differences in the fatty ester profiles between patients with and without CVD, especially in relation to fatty acyl carnitines. These carriers transport fatty acyl groups across the inner membrane of mitochondria to synthesize fatty acids, a critical process in energy metabolism and β-oxidation [48, 49]. Previous studies have shown that alterations to acyl-carnitine metabolism are associated with brain volume changes and cognitive impairment in symptomatic stages of AD [50-52]. Such alterations have also been related to cardiovascular function, such as impaired myocardial relaxation and deteriorated left atrial function, and with an increased risk of recurrent cardiovascular events [53, 54]. Here, we found lower levels of several unsaturated fatty acids in patients with DAT and CVD relative to patients with DAT alone, including octadecenoic (18:1), eicosatrienoic (20:3), arachidonic acid (C20:4) or docosahexaenoic acid (C22:6). Considering that a reduction in these compounds is related to both DAT and CVD, we hypothesize that alterations in these metabolic pathways may have a synergistic effect, favoring the co-existence of DAT and CVD. In addition, we found other alterations to different species of acyl-carnitines, 3 of which increased and 3 decreased in patients with DAT and CVD relative to patients with DAT alone. This result highlights the importance of studying fatty acid metabolism in a comprehensive way, as different species may fulfil different roles in the development of CVD in DAT patients.

Differences in sterol lipids were also found, mainly steroids (all of which were diminished in subjects with DAT and CVD relative to patients with DAT alone) and bile acids (3 decreased and 1 increased in subjects with DAT and CVD relative to patients with DAT alone). Brain steroids are associated with AD as they offer neuroprotection against pathogenic DAT factors like Aβ and against tau pathology, mitochondrial impairment, neuroinflammation, impaired neurogenesis and memory loss [55], consistent with our results. Moreover, several studies associated the decline of steroids with a higher risk of cardiovascular events and mortality [56-59]. Regarding bile acids, increasing evidence suggests AD pathophysiology is influenced by primary and secondary bile acids [60-63]. We found lower levels of chenodeoxycholic acid, a primary bile acid synthetized in the liver that ameliorates AD neurotoxicity, neuroinflammation and cognitive deterioration [64]. Moreover, alterations also appear in the amounts of secondary bile acids, i.e. those resulting from the action of the gut microbiome [65]. Bile acids have been widely studied in relation to the cardiovascular system since the Farnesoid X-activated receptor, a bile acid receptor mainly expressed in the liver and intestine, was identified in cardiomyocytes, endothelium and vascular smooth muscle cells [66, 67]. Nevertheless, the mechanisms underlying the activity of bile acids are not clear. Indeed, both beneficial and detrimental effects on heart function have been reported [68, 69], possibly because the bile acid pool is more important that the actual concentration of any one compound itself [70]. Together, these results show that reduced steroids levels may be detrimental for both brain and cardiovascular function, with low steroid levels related to a higher risk of developing CVD. The results highlight the importance of studying the bile acid pool as opposed to individual compounds. Importantly, agents that sequester bile acids have been approved by the Food and Drug Administration (FDA) and are used as adjuvant therapy for hypercholesterolemia to complement exercise and dietary changes. Further research in this field may be particularly relevant, not only to better understand the molecular mechanisms involved but also, from a therapeutic point of view.

Metabolomics studies on the blood and brains of patients with AD found altered levels of glycerolipids and glycerophospholipids [71-73]. Moreover, there is evidence that oral supplementation with glycerophospholipids can improve cognitive performance, not only in animal models but also in humans with varying degrees of cognitive function [74]. Our data reflect differences in glycerolipids and glycerophospholipids in patients with DAT with and without CVD, suggesting their implication in maintaining cardiac function. We found that all the glycerolipids examined decreased in patients with CVD while 2 glycerophospholipids diminished and 4 increased in those patients. It is clear that glycerolipids and glycerol-phospholipids play crucial roles in cardiovascular health by influencing lipid metabolism, inflammation and metabolic signaling [75]. Triglycerides are among the best studied glycerolipids as they are the most abundant class. Nevertheless, their contribution to the development of CVD is still unclear due to the conflicting results of clinical trials that aim to lower triglycerides [76, 77]. By contrast, genetic evidence supports a causal influence of triglycerides in the development of CAD [78]. This inconsistency may be due to the large number of functionally diverse triglycerides species in circulation, these having varied effects on CVD risk as demonstrated in high-resolution lipidomics studies [79-81]. Our results support these observations, and, in addition, they highlight the importance of studying the specific role of different glycerolipid and glycerophospholipid species in a cohort of patients with CVD in the context of DAT.

Importantly, to obtain a complete image of lipid metabolism it is necessary to evaluate the proteins implicated in these metabolic processes and identify the underlying causal mechanisms that link genotype to phenotype. The rapid technological advances in large-scale ‘omics’ studies are generating vast amounts of data and complex datasets that provide more detailed information on the intricate biological systems within living organisms. Multi-omics integration is emerging as a way of deepening biological understanding, but it faces new limitations and challenges. The use of different omics technologies, with different laboratory techniques results in datasets that can vary in formats, complexity, and dimensionality or scale even others. In addition, the integration of multiple omics datasets leads to a significant increase in the number of variables and is necessary to remove irrelevant or noisy variables. To integrate this new data into current knowledge frameworks requires network-based approaches to help understand the data, new bioinformatics tools that are available to the scientific community [20, 22]. With that in mind, here we integrated the results of a metabolomics and proteomics analysis through OmicsNet (www.omicsnet.ca), a web-based tool that can rapidly generate meaningful information in a multi-omics context to facilitate hypothesis generation and provide mechanistic insights [22].

From our untargeted proteomic study, the bioinformatics tools employed extracted 2 proteins that may serve as biomarkers of CVD in the DAT population: ABCA1 and APOH. These proteins were verified in a new, independent cohort of 28 patients grouped in 4 groups as follows: healthy subjects, patients with CVD, patients with DAT and patients with DAT and CVD. By verifying the potential biomarkers using this approach, we were able to distinguish if the differences observed in the discovery phase were typical of CVD in the context of DAT. Both proteins were verified through orthogonal techniques and were positively correlated with p-Tau(181), a classic hallmark of AD. ABCA1 is a transmembrane protein that transports cholesterol, phospholipids and other lipids to apolipoprotein carriers, and it is essential to sustain cholesterol homeostasis [82]. This protein is expressed strongly in the liver, brain, intestine, and macrophages, and it has been widely studied in AD due to its protective role [83]. It seems clear that ABCA1 upregulation can positively affect APOE lipidation, insulin sensitivity, peripheral vascular and BBB integrity, and anti-inflammatory signaling [84]. Several studies have focused on ABCA1 as a therapeutic target, trying to enhance its expression or activity [85] and producing promising results in preclinical models. Regarding CVD, low levels or defective ABCA1 results in reduced serum levels of HDL cholesterol, deposition of cholesterol in arteries and an increased risk of early onset CVD [83]. However, there is little information on ABCA1 levels in plasma or serum due to its transmembrane nature. Nevertheless, given its transmembrane nature, ABCA1 can be loaded into exosomal membranes that can undergo budding and it was recently proposed that exosomal ABCA1 levels are elevated in the serum of patients with AD [86]. Moreover, exosomes containing ABCA1 are rich in Aβ42, total Tau and p-Tau(181) [87]. Our data suggest that ABCA1 fulfils an important role in the shared mechanisms of CVD and DAT, also suggesting that regulating exosomal turnover may be critical to protect against cardiovascular damage.

The other protein that stood out after the integrated ‘omics’ analysis is APOH, also known as beta-2-glycoprotein 1. APOH is a multifunctional glycoprotein expressed strongly in the liver, endothelial cells, lymphocytes, astrocytes and neurons [88, 89], and it is associated with lipid metabolism, coagulation, apoptosis, inflammation and atherogenesis [90, 91]. During lipid metabolism, APOH binds to several lipoproteins and it stimulates lipoprotein lipase, which participates in triglyceride metabolism and cholesterol transport [92]. There is little information about this protein in AD, although its levels have been suggested to be associated with this pathology, as well as with moderate cognitive impairment and cognitive aging [93]. In terms of the cardiovascular system, APOH has been detected in the sub-endothelial and intima-media regions in arteriosclerosis [94, 95], and it has been associated with adverse health outcomes [88]. In addition, alterations to APOH levels lead to gut microbiome dysregulation and therefore, to impaired bile acid metabolism [96], as seen here in our metabolomics data. Taken together, our results suggest that APOH metabolism is strongly dysregulated in patients with DAT and CVD, with the consequent increase in cardiovascular risk. In terms of the mechanisms involved, our metabolomics results suggest that both glycerolipid and bile acids metabolism could be implicated, emphasizing the relevance of the gut microbiome in human health.

The main limitation of this study is the relatively small sample size, as well as the presence of comorbidities. Although we excluded patients with diabetes, hypertension or dyslipidemia could not be avoided due to their elevated prevalence in patients of advanced age. In an attempt to minimize the effect of these factors, subjects belonging to the different study groups were matched. Nevertheless, studies on larger cohorts will be needed to validate the molecular profiles identified and to further understand the effect of lipid metabolism in the course of cardiovascular disease in the context of AD. This would allow a stratification of the patients according to their comorbidities, which is crucial to understand the synergistic effect that are taking place in elderly population. This phase would represent another step in the search for new biomarkers with a practical clinical application. It is important to continue these studies with an analytical validation phase, which includes, apart from a larger cohort, the use of techniques suitable for the large-scale application of the biomarkers or the development of new ones that could allow the effective implementation in the clinical routine. On the other hand, for a new biomarker to be used, a clinical validation phase is necessary, including rigorous testing under clinical environment and taking into account the cost-benefit assessment compared with other methods already in use.

In conclusion, here we have delved deeper into the relationship between the heart and brain by studying a cohort of individuals with DAT classified according to the presence or absence of CVD. We found alterations to pathways previously implicated separately in those pathologies and significantly, we found that these alterations may be exacerbated in patients in whom both AD and CVD co-exist. This information could be crucial to identify patients at early stages of cardiovascular deterioration who are at high risk of developing AD. This possibility will enable patient interventions to be selected in a personalized manner, employing preventive therapies to slow down cognitive impairment and cardiovascular damage. While traditional lipid profiling measures HDL, LDL, triglycerides and total cholesterol, it is clear that more detailed profiles are needed. Data must be incorporated in other molecular species and proteins that influence the course of DAT and that shed more light on the events driving CVD in the context of DAT. Only in this way can advances be made towards precision medicine, as individuals with different molecular profiles can respond differently to the same management strategies.

## Supplementary Materials

The Supplementary data can be found online at: www.aginganddisease.org/EN/10.14336/AD.2024.0434.
