# Supplementary material for: Dysregulation of Lipid Metabolism Serves as A Link Between Alzheimer’s and Cardiovascular Disease, As Witnessed in A Cross-Sectional Study
Source: Aging Dis. 2024 May 31;16(3):1769–84. doi: 10.14336/AD.2024.0434 (PMC12096944; doi:10.14336/AD.2024.0434)
Supplement: Supplementary file 1 — The Supplementary data can be found online at: www.aginganddisease.org/EN/10.14336/AD.2024.0434. [file AD-16-3-1769-s.pdf]

SUPPLEMENTARY DATA

Dysregulation of Lipid Metabolism Serves as A Link Between Alzheimer's and Cardiovascular Disease, As Witnessed in A Cross-Sectional Study

Laura Mourino-Alvarez, Cristina Juarez-Alia, Tamara Sastre-Oliva, Inés Perales-Sánchez, German Hernandez-Fernandez, Eduardo Chicano-Galvez, Ángela Peralbo-Molina, Felipe Madruga, Emilio Blanco-Lopez, Teresa Tejerina, María G. Barderas

© 2024. Mourino-Alvarez Let al. Published online at http://www.aginganddisease.org/EN/10.14336/AD.2024.0434

SUPPLEMENTARY DATA

Supplementary Table 1. Peak List Used for Mass Calibration.

Calibrant name	m/z	z	Ion Formula
Na(NaCOOH)1	90.9766	1+	Na(NaCOOH)1
Na(NaCOOH)2	158.9641	1+	Na(NaCOOH)2
Na(NaCOOH)3	226.9515	1+	Na(NaCOOH)3
Na(NaCOOH)4	294.9389	1+	Na(NaCOOH)4
Na(NaCOOH)5	362.9264	1+	Na(NaCOOH)5
Na(NaCOOH)6	430.9139	1+	Na(NaCOOH)6
Na(NaCOOH)7	498.9014	1+	Na(NaCOOH)7
Na(NaCOOH)8	566.8889	1+	Na(NaCOOH)8
Na(NaCOOH)9	634.8764	1+	Na(NaCOOH)9
Na(NaCOOH)10	702.8639	1+	Na(NaCOOH)10
Na(NaCOOH)11	770.8514	1+	Na(NaCOOH)11
Na(NaCOOH)12	838.8389	1+	Na(NaCOOH)12
Na(NaCOOH)13	906.8264	1+	Na(NaCOOH)13
Na(NaCOOH)14	974.8139	1+	Na(NaCOOH)14
Na(NaCOOH)15	1042.801	1+	Na(NaCOOH)15
Na(NaCOOH)16	1110.789	1+	Na(NaCOOH)16
Na(NaCOOH)17	1178.776	1+	Na(NaCOOH)17
Na(NaCOOH)18	1246.764	1+	Na(NaCOOH)18
Na(NaCOOH)19	1314.751	1+	Na(NaCOOH)19
Na(NaCOOH)20	1382.739	1+	Na(NaCOOH)20
Na(NaCOOH)21	1450.726	1+	Na(NaCOOH)21
Na(NaCOOH)22	1518.714	1+	Na(NaCOOH)22
HCOO(NaCOOH)1	112.9856	1-	HCOO(NaCOOH)1
HCOO(NaCOOH)2	180.9731	1-	HCOO(NaCOOH)2
HCOO(NaCOOH)3	248.9605	1-	HCOO(NaCOOH)3
HCOO(NaCOOH)4	316.9479	1-	HCOO(NaCOOH)4
HCOO(NaCOOH)5	384.9353	1-	HCOO(NaCOOH)5
HCOO(NaCOOH)6	452.9227	1-	HCOO(NaCOOH)6
HCOO(NaCOOH)7	520.9101	1-	HCOO(NaCOOH)7
HCOO(NaCOOH)8	588.8975	1-	HCOO(NaCOOH)8
HCOO(NaCOOH)9	656.8849	1-	HCOO(NaCOOH)9
HCOO(NaCOOH)10	724.8723	1-	HCOO(NaCOOH)10
HCOO(NaCOOH)11	792.8597	1-	HCOO(NaCOOH)11
HCOO(NaCOOH)12	860.8471	1-	HCOO(NaCOOH)12
HCOO(NaCOOH)13	928.8345	1-	HCOO(NaCOOH)13
HCOO(NaCOOH)14	996.8219	1-	HCOO(NaCOOH)14
HCOO(NaCOOH)15	1064.809	1-	HCOO(NaCOOH)15
HCOO(NaCOOH)16	1132.797	1-	HCOO(NaCOOH)16
HCOO(NaCOOH)17	1200.784	1-	HCOO(NaCOOH)17
HCOO(NaCOOH)18	1268.772	1-	HCOO(NaCOOH)18
HCOO(NaCOOH)19	1336.759	1-	HCOO(NaCOOH)19
HCOO(NaCOOH)20	1404.746	1-	HCOO(NaCOOH)20
HCOO(NaCOOH)21	1472.734	1-	HCOO(NaCOOH)21

Supplementary Table 2. Peak List Used for Mobility Calibration.

Calibrant formula	m/z	Charge	Mobility
C5H12NO2	118.0863	1+	0.545
C6H19N3O6P3	322.0481	1+	0.736
C12H19F12N3O6P3	622.029	1+	0.991
C18H19F24N3O6P3	922.0098	1+	1.199
C24H19F36N3O6P3	1221.9906	1+	1.393
C30H19F48N3O6P3	1521.9715	1+	1.568
C36H19F60N3O6P3	1821.9523	1+	1.741
C42H19F72N3O6P3	2121.9331	1+	1.9
C48H19F84N3O6P3	2421.914	1+	2.05
C54H19F96N3O6P3	2721.8948	1+	2.192

© 2024. Mourino-Alvarez Let al. Published online at http://www.aginganddisease.org/EN/10.14336/AD.2024.0434

C2F3O2	112.9856	1-	0.484
C6HF9N3O	301.9981	1-	0.669
C12HF21N3O	601.979	1-	0.882
C20H18F27N3O8P3	1033.9881	1-	1.258
C26H18F39N3O8P3	1333.9689	1-	1.407
C32H18F51N3O8P3	1633.9498	1-	1.58
C38H18F63N3O8P3	1933.9306	1-	1.748
C44H18F75N3O8P3	2233.9115	1-	1.89
C50H18F87N3O8P3	2533.8923	1-	2.051
C56H18F99N3O8P3	2833.8731	1-	2.15

Supplementary Table 3. Optimized DIA-PASEF method for protein identification and quantification.

MS type	Cycle ID	1/K0 begin (VS/cm2)	1/K0 end (VS/cm2)	Start mass (m/z)	End mass (m/z)
MS1	0	-	-	-	-
PASEF	1	0.9	1.3	657.84	682.89
PASEF	1	0.7	0.9	352.54	414.21
PASEF	2	0.92	1.3	682.89	709.81
PASEF	2	0.7	0.92	414.21	444.75
PASEF	3	0.93	1.3	709.81	735.87
PASEF	3	0.7	0.93	444.75	473.61
PASEF	4	0.95	1.3	735.87	766.37
PASEF	4	0.7	0.95	473.61	499.78
PASEF	5	0.96	1.3	766.37	796.76
PASEF	5	0.7	0.96	499.78	518.76
PASEF	6	0.97	1.3	796.76	825.73
PASEF	6	0.7	0.97	518.76	540.79
PASEF	7	0.98	1.3	825.73	858.79
PASEF	7	0.7	0.98	540.79	560.82
PASEF	8	0.99	1.3	858.79	895.95
PASEF	8	0.7	0.99	560.82	579.29
PASEF	9	1.0	1.3	895.95	940.77
PASEF	9	0.7	1	579.29	598.8
PASEF	10	1.01	1.3	940.77	984.49
PASEF	10	0.7	1.01	598.8	616.33
PASEF	11	1.03	1.3	984.49	1067.06
PASEF	11	0.7	1.03	616.33	638.29
PASEF	12	1.05	1.3	1067.06	1199.55
PASEF	12	0.7	1.05	638.29	657.84

Supplementary Table 4. Western blot conditions including blocking solution, primary and secondary antibodies.

Antigen	Blocking solution	Primary antibody, dilution	Secondary antibody, dilution
ABCA1	5% nonfat milk-TBS 0.1% Tween-20	ab307536 (Abcam), 1:500	#7074P2 (Cell signaling), 1:1000
APOH	7.5% nonfat milk-PBS 0.05% Tween-20	ab108348 (Abcam), 1:5000	ab205718 (Abcam), 1:2000
Phosphor-Tau(Thr181)	5% nonfat milk-TBS 0.1% Tween-20	#12885 (Cell signaling), 1:250)	#7074P2 (Cell signaling), 1:1000

© 2024. Mourino-Alvarez Let al. Published online at http://www.aginganddisease.org/EN/10.14336/AD.2024.0434

SUPPLEMENTARY DATA

Supplementary Table 5. Identified metabolites during the DIA-PASEF experiment, including the molecular formula, Pubchem identifier and fold change (FC, DAT+CVD/DAT).

Mode	Feature	Name	Molecular formula	Pubchem	FC	Category	Category II	Category III
Pos	136.03864 Da 120.28 s	Hypoxanthine	C5H4N4O	135398638	1,7327	Organoheterocyclic compounds	Imidazopyrimidines	Purines and purine derivatives
Pos	143.09472 Da 82.46 s	Proline Betaine	C7H13NO2	115244	3,7202	Organic acids and derivatives	Carboxylic acids and derivatives	Amino acids, peptides, and analogues
Pos	161.10519 Da 78.43 s	L-Carnitine	C7H15NO3	10917	1,2089	Organic nitrogen compounds	Quaternary ammonium salts	Carnitines
Neg	168.02817 Da 82.66 s	3-hydroxyxhantine	C5H4N4O3	83520	1,8815	Organoheterocyclic compounds	Imidazopyrimidines	Purines and purine derivatives
Pos	168.02834 Da 120.01 s	uric acid	C5H4N4O3	1175	1,8278	Organoheterocyclic compounds	Imidazopyrimidines	Purines and purine derivatives
Neg	188.01420 Da 414.14 s	p-Cresol sulfate	C7H8O4S	4615423	1,5806	Organic acids and derivatives	Organic sulfuric acids and derivatives	Arylsulfates
Neg	204.00897 Da 392.81 s	3-Methoxyphenol sulfate	C7H8O5S	59815435	0,57855	Benzenoids	Phenols	Methoxyphenols
Neg	205.07364 Da 471.33 s	Indolelactic acid	C11H11NO3	92904	1,4608	Organoheterocyclic compounds	Indoles and derivatives	Indolyl carboxylic acids and derivatives
Neg	213.00932 Da 357.69 s	Indoxyl sulfate (95.566%)	C8H7NO4S	10258	1,8603	Organic acids and derivatives	Organic sulfuric acids and derivatives	Arylsulfates
Neg	214.02959 Da 524.41 s	Chavicol hydrogen sulfate	C9H10O4S	66633409	6,0489	Organic acids and derivatives	Organic sulfuric acids and derivatives	Arylsulfates
Neg	254.22445 Da 1005.30 s	Palmitoleic acid	C16H30O2	445638	0,68467	Lipids and lipid-like molecules	Fatty Acyls [FA]	Fatty Acids and Conjugates
Neg	264.11089 Da 386.53 s	Alpha-N-phenylacetyl-L-glutamine	C13H16N2O4	92258	2,107	Organic acids and derivatives	Carboxylic acids and derivatives	Amino acids, peptides, and analogues
Neg	272.23499 Da 992.42 s	FA 16:0;(2OH)	C16H32O3	92836	0,71949	Lipids and lipid-like molecules	Fatty Acyls	Fatty Acids and Conjugates
Neg	280.24018 Da 1020.63 s	FA 18:2 (Octadecadienoic acid)	C18H32O2		0,5385	Lipids and lipid-like molecules	Fatty Acyls	Fatty Acids and Conjugates
Neg	282.25593 Da 1075.69 s	Octadecenoic acid	C18H34O2		0,62521	Lipids and lipid-like molecules	Fatty Acyls	Fatty Acids and Conjugates; Octadecanoids
Pos	285.19369 Da 529.52 s	octenoyl-carnitine	C15H27NO4	129692230	2,3089	Lipids and lipid-like molecules	Fatty Acyls	Fatty esters
Pos	287.20961 Da 584.47 s	Octanoyl-L-carnitine	C15H29NO4	11953814	0,61191	Lipids and lipid-like molecules	Fatty Acyls	Fatty esters
Neg	292.09098 Da 128.15 s	4-amino-N-{2-[(4-nitrophenyl) amino]ethyl} -1,2,5-oxadiazole-3-carboxamide	C11H12N6O4	2861367	0,8802	Undetermined		
Neg	304.24025 Da 1005.15 s	Arachidonic acid	C20H32O2	444899	0,30293	Lipids and lipid-like molecules	Fatty Acyls	Fatty Acids and Conjugates

© 2024. Mourino-Alvarez Let al. Published online at http://www.aginganddisease.org/EN/10.14336/AD.2024.0434

SUPPLEMENTARY DATA

Neg	306.25582 Da 1054.65 s	8,11,14-Eicosatrienoic acid	C20H34O2	5280581	0,51502	Lipids and lipid-like molecules	Fatty Acyls	Fatty Acids and Conjugates
Neg	306.25600 Da 1037.79 s	FA 20:3 (Eicosatrienoic acid)	C20H34O2		0,5028	Lipids and lipid-like molecules	Fatty Acyls	Fatty Acids and Conjugates
Pos	315.24097 Da 676.94 s	decanoylcarnitine	C17H33NO4	11953821	0,56844	Lipids and lipid-like molecules	Fatty Acyls	Fatty esters
Neg	328.24027 Da 988.77 s	Docosahexaenoic acid	C22H32O2	5353594	0,70011	Lipids and lipid-like molecules	Fatty Acyls	Fatty Acids and Conjugates
Neg	336.05643 Da 120.25 s	1,3-Diazidopropan-2-yl-3,5-dinitrobenzoate	C10H8N8O6	129831155	1,654	Undetermined		
Pos	343.27264 Da 763.81 s	Lauroylcarnitine	C19H37NO4	168381	0,70103	Lipids and lipid-like molecules	Fatty Acyls	Fatty esters
Pos	359.31265 Da 1105.85 s	(2r)-5-Amino-2-[(r)-3-hydroxy tetradecanoylamino] pentan-1-ol amine	C19H41N3O3	87421561	1,2202	Undetermined		
Neg	366.27725 Da 919.72 s	FAHFA 22:2			0,62287	Lipids and lipid-like molecules	Fatty Acyls	Fatty esters
Neg	368.16621 Da 596.62 s	DHEA sulfate	C19H28O5S	12594	0,3466	Lipids and lipid-like molecules	Sterol Lipids	Steroid conjugates; Steroids
Neg	370.18230 Da 608.64 s	Epiandrosterone sulfate	C19H30O5S	9929317	0,22124	Lipids and lipid-like molecules	Sterol Lipids	Steroids
Neg	372.22841 Da 1004.81 s	Desoxycorticosterone acetate	C23H32O4	5952	0,52903	Lipids and lipid-like molecules	Sterol Lipids	Steroids
Neg	384.16175 Da 523.66 s	3b,16a-Dihydroxyandrostenone sulfate	C19H28O6S	20848951	0,49062	Lipids and lipid-like molecules	Sterol Lipids	Steroid conjugates
Neg	392.29090 Da 799.26 s	Chenodeoxycholic acid	C24H40O4	10133	0,68898	Lipids and lipid-like molecules	Sterol Lipids	Bile acids and derivatives
Neg	398.21312 Da 625.20 s	Epipregnanolone sulphate	C21H34O5S	73755084	0,47894	Lipids and lipid-like molecules	Sterol Lipids	Steroids
Pos	414.20444 Da 723.94 s	estriol triacetate	C24H30O6	15125811	9,469	Undetermined		
Pos	423.33619 Da 892.99 s	Linoleyl-carnitine	C25H46NO4	6450015	1,4388	Lipids and lipid-like molecules	Fatty Acyls	Fatty esters; Fatty Acids and Conjugates
Pos	425.35058 Da 956.70 s	oleyl-L-carnitine	C25H48NO4	46907933	1,3446	Lipids and lipid-like molecules	Fatty Acyls	Fatty esters
Neg	438.29859 Da 799.39 s	Arenolide	C25H42O6	10741676	0,69974	Lipids and lipid-like molecules	Polyketides	Macrolides and lactone polyketides
Neg	446.33990 Da 1049.73 s	FAHFA 28:4			0,51532	Lipids and lipid-like molecules	Fatty Acyls	Fatty esters
Pos	447.29751 Da 675.54 s	7-Oxoglycochenodeoxycholic acid	C26H41NO5	3086041	0,26295	Lipids and lipid-like molecules	Sterol Lipids	Steroid conjugates; Bile acids and derivatives
Neg	449.31462 Da 642.22 s	Glycochenodeoxycholic acid	C26H43NO5	12544	6,1032	Lipids and lipid-like molecules	Sterol Lipids	Steroid conjugates
Neg	460.11950 Da 120.00 s	[4-[[4-Bis(ethenyl)phosphoryloxyphenyl]	C23H26O6P2	57815710	1,182	Undetermined		

© 2024. Mourino-Alvarez Let al. Published online at http://www.aginganddisease.org/EN/10.14336/AD.2024.0434

		methyl]phenyl] bis(prop-2-enyl) phosphate						
Pos	468.38059 Da 1054.55 s	DAG 25:1			0,61434	Lipids and lipid-like molecules	Glycerolipids	Diradylglycerols
Neg	470.23434 Da 617.24 s	ST 24:2;O3;S	C24H40O6S	451489	0,53994	Lipids and lipid-like molecules	Sterol Lipids	Steroid conjugates
Neg	472.24956 Da 604.48 s	Chenodeoxycholic acid sulfate	C24H40O7S	20849194	6,6139	Lipids and lipid-like molecules	Sterol Lipids	Steroid conjugates
Pos	477.28557 Da 886.73 s	LPE 18:2	C23H44NO7P	52925130	1,3817	Lipids and lipid-like molecules	Glycerophospholipids	Glycerophosphoethanolamines
Pos	479.30132 Da 998.30 s	LPE 18:1	C23H46NO7P	53480925	1,3456	Lipids and lipid-like molecules	Glycerophospholipids	Glycerophosphoethanolamines
Neg	486.17415 Da 489.39 s	[(3S,4S,5S)-5-[(2S,3R,4S,5S,6R)-4,5- dihydroxy-6-(hydroxymethyl)-2- (4-hydroxyphenoxy) oxan-3-yl]oxy-3,4-dihydroxyoxolan-3-yl] methyl (E)-2-methylbut-2-enoate	C22H30O12	163195342	1,7763	Undetermined		
Pos	499.26969 Da 819.11 s	PE(0:0/20:5)	C25H42NO7P	52925146	1,5378	Lipids and lipid-like molecules	Glycerophospholipids	Glycerophosphoethanolamines
Pos	501.28556 Da 874.17 s	LysoPE(20:4(8Z,11Z,14Z,17Z)/0:0)	C25H44NO7P	53480952	0,81686	Lipids and lipid-like molecules	Glycerophospholipids	Glycerophosphoethanolamines
Pos	518.39519 Da 1118.98 s	DG 29:4			0,55327	Lipids and lipid-like molecules	Glycerolipids	Diradylglycerols
Pos	520.41081 Da 1053.50 s	DG 29:3			0,55039	Lipids and lipid-like molecules	Glycerolipids	Diradylglycerols
Neg	529.27109 Da 563.57 s	Glyco 3a-sulfate-7a-OH-5b-cholanic acid	C26H43NO8S	21125002	4,8375	Lipids and lipid-like molecules	Sterol Lipids	Steroid conjugates
Neg	529.27139 Da 624.08 s	ST 24:1;O3;G;S	C26H43NO7S	443113	0,51868	Lipids and lipid-like molecules	Sterol Lipids	Steroid conjugates; Bile acids and derivatives
Neg	541.33849 Da 930.67 s	1-(2-Methoxy-octadecanyl)-sn-glycero-3-phosphoserine	C25H52NO9P	137323953	1,1615	Lipids and lipid-like molecules	Glycerophospholipids	Glycerophosphoserines
Pos	543.33282 Da 1282.77 s	LPC 20:4	C28H50NO7P	53480469	0,68113	Lipids and lipid-like molecules	Glycerophospholipids	Glycerophosphocholines
Neg	544.28826 Da 509.74 s	Cortol-3-glucuronide	C27H44O11	44144542	1,9573	Undetermined		
Pos	554.04619 Da 394.01 s	[(2R,3S,4R,5R)-3,4-dihydroxy-5-[4-[(4-nitrophenyl)methoxyamino]-2-oxopyrimidin-1-yl]oxolan-2-yl]methyl phosphono hydrogen phosphate	C16H20N4O14P2	90666793	0,63967	Undetermined		
Pos	557.46495 Da 1069.87 s	LDGTS 22:0			0,48913	Organic acids and derivatives	Carboxylic acids and derivatives	Amino acids, peptides, and analogues
Neg	568.32475 Da 689.65 s	beta-D-glucuronide			0,4095	Organic oxygen compounds	Carbohydrates and carbohydrate conjugates	

© 2024. Mourino-Alvarez Let al. Published online at http://www.aginganddisease.org/EN/10.14336/AD.2024.0434

SUPPLEMENTARY DATA

Pos	584.26330 Da 1106.25 s	Bilirrubin	C33H36N4O6	5280352	1,6332	Organoheterocyclic compounds	Tetrapyrroles and derivatives	Bilirubins
Neg	584.32001 Da 635.53 s	Cholic acid glucuronide	C30H48O11	21252309	2,7905	Lipids and lipid-like molecules	Sterol Lipids	Steroid conjugates; Bile acids and derivatives
Neg	606.16383 Da 85.59 s	3-[8-(3-Carboxyphenyl)-6-(2-hydroxyphenyl)-1,3,7,9-tetraoxo-3a,4,6,6a,9a,10,10a,10b-octahydroisoindolo [5,6-e]isoindol-2-yl]benzoic acid	C34H26N2O9	3621977	0,88221	Undetermined		
Pos	611.51207 Da 1113.93 s	LDGTS 26:1	C36H69NO6	138225477	0,62604	Organic acids and derivatives	Carboxylic acids and derivatives	Amino acids, peptides, and analogues
Pos	616.46921 Da 1115.51 s	TG 8:0_11:0_16:4			0,56874	Lipids and lipid-like molecules	Glycerolipids	Triradylglycerols
Neg	641.34205 Da 581.21 s	SHexCer 23:1;3O	C29H55NO12S	165228504	0,44322	Undetermined		
Neg	650.12979 Da 119.94 s	(4-nitrophenyl)methyl (4S,5R,6S)-3-[7-[2-(dimethylsulfamoylamino) ethylsulfanyl]imidazo[5,1-b][1,3]thiazol-2-yl]-6-[(1R)-1-hydroxyethyl]-4-methyl-7-oxo-1-azabicyclo[3.2.0]hept-2-ene-2-carboxylate	C26H30N6O8S3	91217185	1,2905	Undetermined		

© 2024. Mourino-Alvarez Let al. Published online at http://www.aginganddisease.org/EN/10.14336/AD.2024.0434
